# Establishing an Essential Dataset for Trauma Registry in LMICs: Insights From a Delphi Survey

**DOI:** 10.1002/wjs.70009

**Published:** 2025-07-16

**Authors:** Theresa Farhat, Steven Di Marco, Gabriela Sanchez, Samjhana Basnet, Ibrahima Konate, Vitaliy Krylyuk, Respicious Boniface, Victoria Munthali, Tarek Razek, Jeremy Grushka, Dan Deckelbaum

**Affiliations:** ^1^ Centre for Global Surgery McGill University Health Centre Montréal Canada; ^2^ Department of General Practice and Emergency Medicine Dhulikhel Hospital Kathmandu University Hospital Dhulikhel Nepal; ^3^ Department of Surgery Gaston Berger University of Saint‐Louis Saint‐Louis Senegal; ^4^ Training Centre at Kyiv Hospital for Emergency Medicine Kyiv Ukraine; ^5^ Injury Control Centre Tanzania Muhimbili Orthopedic Institute Dar es Salaam Tanzania

**Keywords:** Delphi survey, health system strengthening, low‐ and middle‐income countries (LMICs), trauma care, trauma registry

## Abstract

**Background:**

Injury is a leading cause of morbidity and mortality globally, with 90% of deaths occurring in low‐middle‐income countries (LMICs). Establishing well‐functioning trauma systems is crucial in LMICs, with a trauma registry being an integral component. This study used the Delphi Technique to gather insights from trauma experts on essential data variables for adult trauma registries in LMICs. It aimed to identify critical variables that can improve trauma care in resource‐constrained settings.

**Methods:**

A two‐round Delphi survey was conducted from October 2023 to June 2024, engaging trauma specialists from diverse regions. Experts evaluated variables as essential, optional, or excluded, with a consensus of 70% agreement. Feedback from the first round informed the second round, focusing on variables lacking consensus.

**Results:**

In the first round, 37 variables reached consensus as essential, including demographics, injury‐related data, prehospital information, some clinical assessment variables, injury classification, road traffic accident data, and patient outcome data. The second round identified additional variables and categorized others as optional, including education level, income level, certain advanced imaging modalities, cost of care, and some outcome measures. Birthplace was identified as the only variable for exclusion from the trauma registry.

**Conclusions:**

This study identifies essential elements of a trauma registry in LMICs, leveraging insights from experts experienced in resource‐limited settings. These recommendations ensure relevance and feasibility for implementation. Establishing such a registry is crucial for quality assurance, jurisdictional comparisons, and the foundation of trauma systems.

## Graphical abstract

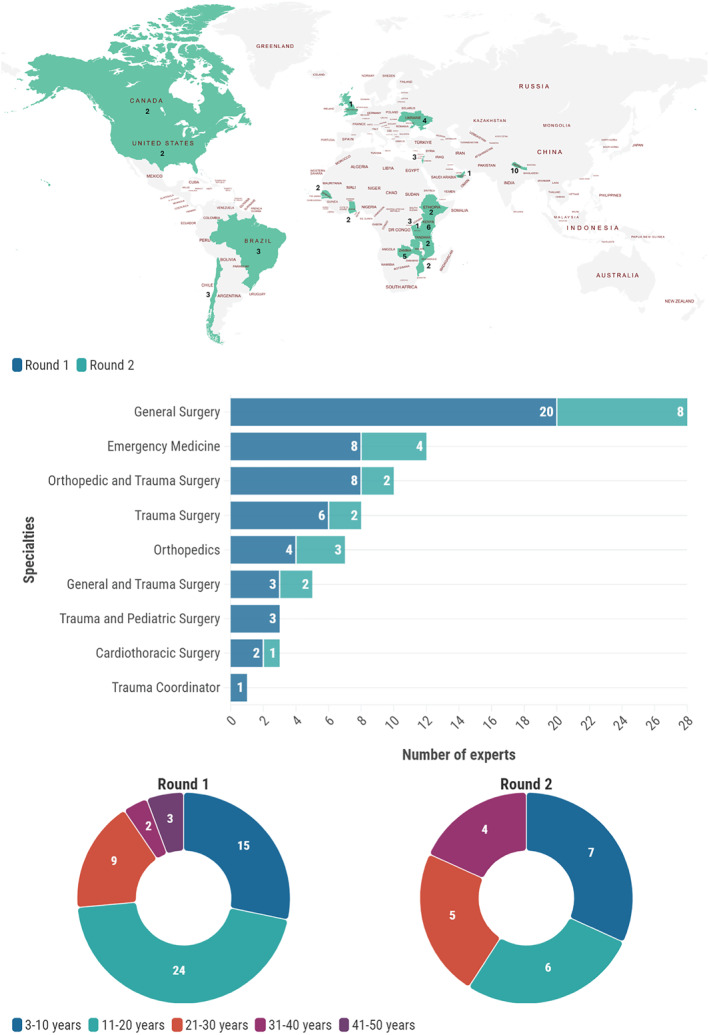

**Abbreviations**
CGS: Centre of Global Surgery
CT: computed tomography
ECG: Electrocardiogram
ER: emergency room
HICs: high‐income countries
ISS: Injury Severity Score
KTS: Kampala Trauma Score
LMICs: low‐ and middle‐income countries
MD: Medical Doctor
MRI: magnetic resonance imaging
NTDB: National Trauma Data Bank
PTSD: post‐traumatic stress disorder
RTA: road traffic accident
RTS: Revised Trauma Score
TQIP: Trauma Quality Improvement Program

## 1 Introduction

Injury is a leading cause of morbidity and mortality globally [1]. Every year, over 4.4 million people around the world die from injuries, accounting for 9% of all deaths [2], and nearly 1 billion people sustain injuries that require some form of healthcare [3]. They are responsible for an estimated 10% of all the years lived with disability [2]. Nearly 90% of injury‐related deaths occur in low‐ and middle‐income countries (LMICs), where inadequate trauma care systems exacerbate the burden of trauma [4]. The mortality from road traffic accidents (RTAs) is 27.5 per 100,000 in LMICs compared to 8.3 per 100,000 in high‐income countries (HICs) [5]. Therefore, establishing well‐functioning trauma systems is crucial in LMICs, with the collection and analysis of injury data in the form of a trauma registry being an integral component.

Trauma registries are comprehensive databases of epidemiological, care process, and outcome data on injured patients [6]. They provide essential evidence for quality improvement, injury prevention, research, and policy development by improving understanding of trauma epidemiology and patient outcomes [6]. However, in most LMICs, trauma registries are either absent or limited in the amount and quality of data they collect due to a lack of funding, specialized personnel, and related infrastructure [7]. There is also a lack of standardized methods for determining what, how, and on whom the data are collected [7, 8]. This makes it challenging to do comparative analyses of trauma care and outcomes to improve the quality of care.

The Centre of Global Surgery (CGS) at the McGill University Health Centre (MUHC) has collaborated with partners from LMICs for over a decade to enhance surgical capacity. These partnerships have offered valuable insights into the needs and obstacles in LMICs, leading CGS to develop a trauma registry based on the essential dataset through cooperative efforts. It consists of de‐identified data elements such as patient demographics, mechanism of injury, injury severity, and outcomes.

This research aimed to validate variables that are essential and feasible to collect for an ideal adult trauma registry dataset in LMICs based on inputs from experts with diverse backgrounds and experience working in LMICs through a Delphi survey. The results highlight a core set of variables for trauma registry data collection, offering a standardized dataset that provides a crucial foundation for accurate and consistent data across trauma systems.

## 2 Methods

### 2.1 Delphi Technique

A Delphi survey was conducted to generate insights from trauma experts in LMICs or experts based in HICs with experience working in LMICs on the essential data variables that should be included in LMICs’ adult trauma registries. The Delphi method builds on the anonymity of participating experts invited to participate in the survey and has proven to be an effective means of reaching expert consensus on different topics in healthcare settings [9].

### 2.2 The Expert Panel

The expert panel included trauma, general, and orthopedic surgeons, as well as emergency physicians with experience working in LMICs in Asia, Latin America, North America, Europe, Africa, and the Middle East, ensuring diverse geographic and clinical perspectives. Participants were selected based on their clinical and leadership roles in trauma care, with nonexperts and individuals lacking LMIC trauma experience excluded. Experts were identified through trauma networks, professional societies, and collaborations.

### 2.3 Delphi Methodology

A two‐round Delphi survey was conducted between October 2023 and June 2024. After conducting an internal pilot test to ensure there were no technical issues and to confirm the clarity of instructions and objectives, 131 experts received invitations via email to fill out the Delphi survey using the web‐based platform online surveys (QuestionPro). Before starting, experts were required to read the consent form, and their participation in the survey was considered implied consent. Clear instructions on the survey's purpose, process, definitions, and consent were provided at the start to ensure consistency and understanding among experts.

*Round 1:* Experts were asked to evaluate each variable for inclusion in adult trauma registries in LMICs, assigning each to one of three categories: Essential—critical for effective trauma care, quality improvement, and registry sustainability and feasible to collect. Optional—useful but not indispensable and therefore recorded only when resources permit. Excluded—unnecessary and thus should not be included in the registry. Experts were also asked to provide comments and suggestions after each section. Reminder emails were sent to nonrespondents and those who did not participate in a round were not eligible to participate in subsequent rounds. Variables were assessed based on the percentage of agreement among experts. Consensus was defined as ≥ 70% agreement on whether a variable should be considered essential, optional, or excluded in the first round.

*Round 2:* This round focused on variables that did not achieve consensus in Round 1. Experts received feedback on Round 1 consensus status and reviewed a revised questionnaire with only variables that did not reach consensus. During this phase, experts reassessed their conclusions considering new information. The goal was to achieve consensus on variables considered “essential” and practical to collect in resource‐limited settings.

### 2.4 Analysis

The percentage distribution for each variable was calculated to determine the level of agreement among participants. Variables that reached a consensus in round 1—defined as ≥ 70% agreement on whether they were essential, optional, or excluded—were removed from the survey. Only variables that did not reach this threshold were carried forward to the second round. In round two, most variables reached consensus. For those that did not achieve 70%, the decision to classify them as essential, optional, or excluded was based on the highest percentage score attained.

### 2.5 Ethical Considerations

The research ethics board of the McGill University Health Centre approved this study (Protocol # 2024‐9686). Starting the survey was considered consent to participate as clearly outlined in the survey instructions.

## 3 Results

### 3.1 Demographic Characteristics of Delphi Survey Participants

**FIGURE 1:**
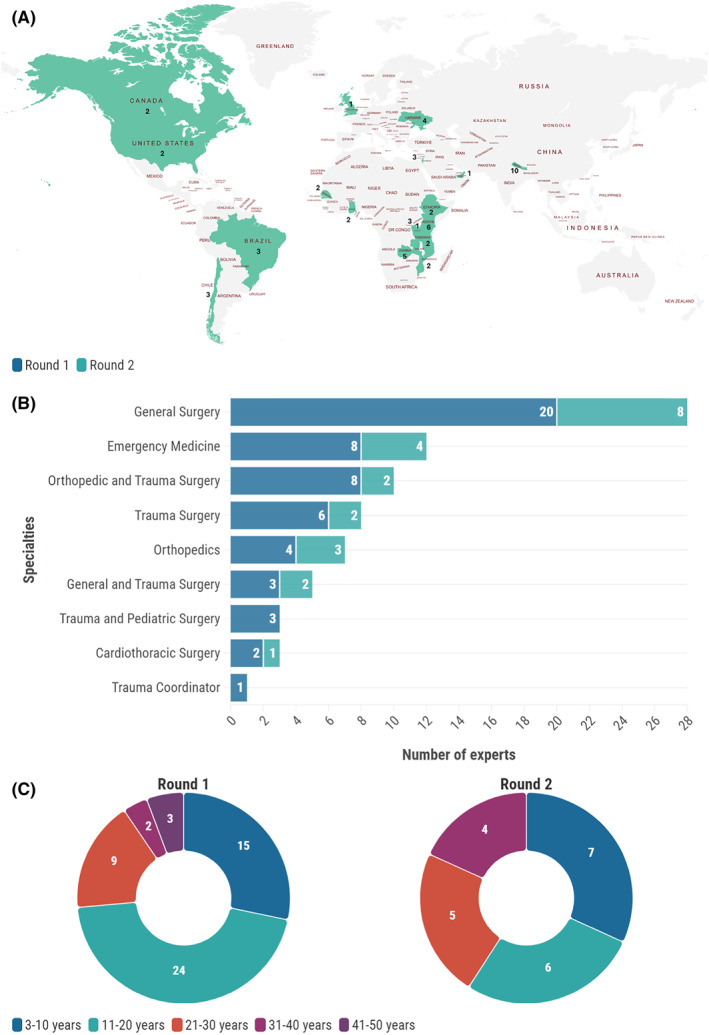
(A) Global distribution of experts who participated in the Delphi survey, highlighting key regions of representation. (B) Breakdown of the specialties of the participating experts. (C) Distribution of experts by years of experience.

The Delphi survey comprised two rounds. In the first round, 42.0% of invited experts (*n* = 55/131) participated, and of those, 40.0% (*n* = 22/55) participated in the second round. Most participants in the first round were men (81.8%, *n* = 45/55) with 11–20 years (43.6%, *n* = 24/55) of professional experience. The two most prevalent specialties were general surgery and emergency medicine, accounting for 15.5% (*n* = 20/55) and 14.5% (*n* = 8/55), respectively. The geographic distribution of experts shows 46.3% from Africa (*n* = 25/54), 25.9% from Asia (*n* = 14/54), 11.1% from South America (*n* = 6/54), 9.3% from Europe (*n* = 5/54), and 7.4% from North America (*n* = 4/54), with higher representation from Nepal (18.2%, *n* = 10/55) and Kenya (10.9%, *n* = 6/55) (Figure 1).

In the second round, 72.7% (*n* = 16/22) of participants were men. The experience was divided into various groups, with 31.8% (7/22) falling into the 3–10‐year category and 27.3% (*n* = 6/22) falling into the 11–20‐year category. The percentage of general surgery specialists rose to 36.4% (*n* = 8/22) of specialists. The geographic distribution remained consistent, with Nepal (13.6%, *n* = 3/22) and Kenya (13.6%, *n* = 3/22) making major contributions (Figure 1).

### 3.2 First‐Round Consensus

**FIGURE 2:**
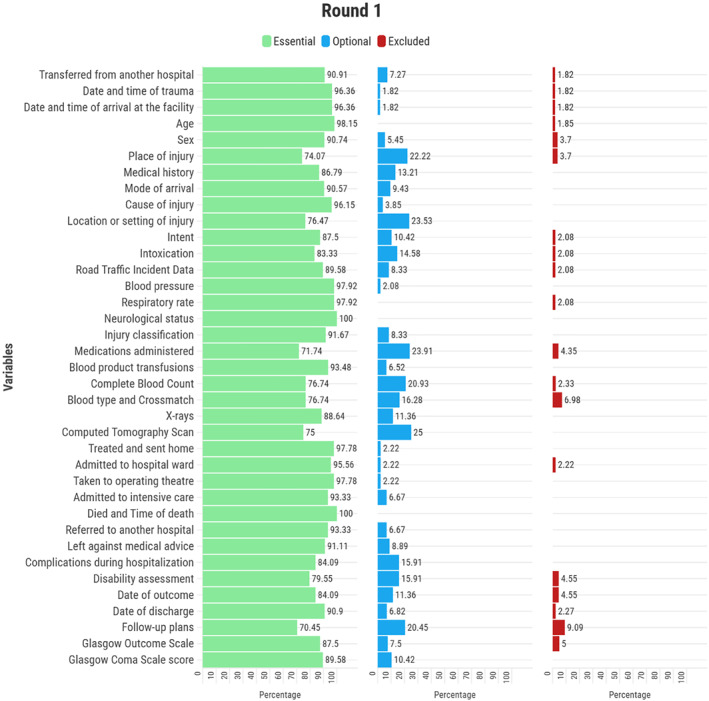
Variables that reached ≥ 70% consensus as essential in LMICs during the first round of the Delphi survey.

In the first round, 37 variables reached consensus as essential components of the adult trauma registry in LMICs (Figure 2). This included demographics such as age (98.1%) and sex (90.7%). Similarly, injury‐related information, such as the date and time of trauma (96.4%), cause of injury (96.1%), intent (87.5%), and location of injury (76.5%), reached consensus as essential variables. Prehospital information, including patient transfer data (90.9%) and mode of arrival (90.6%), was also deemed crucial. Information from before arriving at the hospital, such as if the patient was transferred from another hospital (90.9%) and how they arrived (90.6%), was also deemed crucial for inclusion in the registry.

Clinical assessment variables that reached consensus included arterial blood pressure (97.9%), respiratory rate (97.9%), neurological status (100.0%), presence of serious injuries (93.7%), and Glasgow Coma Scale score (89.6%). In addition, medical history (86.8%) and intoxication level (83.3%) were also considered essential data variables.

The survey participants agreed on including road traffic incident data (89.6%) and injury classification (91.7%). Surgeries, therapeutic procedures, and blood transfusions were also considered important variables of the registry, with percentages of 91.5% and 93.5%, respectively.

Agreement was reached on diagnostic tests, with laboratory tests at 73.9% and imaging modalities at 89.1%. It was also considered necessary to conduct specific tests such as a complete blood count (76.7%), X‐rays (88.6%), CT scan (75.0%), and ultrasound (84.1%).

Patient outcome data, such as whether the patient was treated and sent home (97.8%), admitted to a hospital ward (95.6%), taken to the operating theater (97.8%), admitted to intensive care (93.3%), died (100.0%), referred to another hospital (93.3%), or left against medical advice (91.1%), all reached consensus.

Finally, the participants deemed information regarding complications during hospitalization (84.1%), disability evaluation (79.5%), date of outcome (84.1%), discharge date (90.9%), follow‐up arrangements (70.4%), and Glasgow Outcome Scale (87.5%) to be crucial (Figure 2). No variables reached ≥ 70% consensus for exclusion in the first round.

### 3.3 Second Round Consensus

**FIGURE 3:**
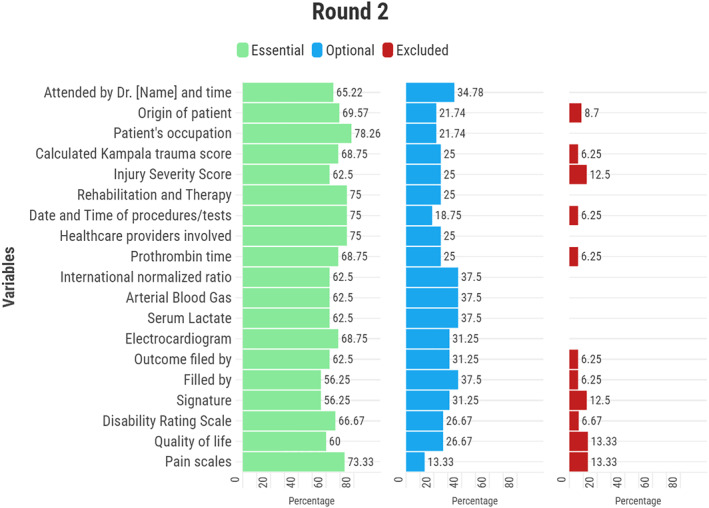
Variables that reached consensus as essential in the second round of the Delphi survey. Options with the highest score, achieving more than 55% agreement, were considered to have reached a consensus.

In the second round of the Delphi survey, several variables were identified as essential for inclusion in the LMIC trauma registry (Figure 3). Options with the highest score, achieving more than 55% agreement, were considered to have reached a consensus. These included the name and time of the doctor in charge (65.2%), the patient's place of origin (69.6%), and their occupation (78.3%). Clinical scoring systems, such as the Kampala Trauma Score (KTS) (68.7%) and the Injury Severity Score (ISS) (62.5%), were also considered crucial. Additionally, information related to rehabilitation and therapy (75.0%), dates and times of procedures and tests (75.0%), healthcare providers and specialists involved (75.0%), and electrocardiogram (ECG) results (68.7%) were considered necessary. Laboratory tests, including prothrombin time (68.7%), international normalized ratio (62.5%), arterial blood gas (62.5%), and serum lactate (62.5%), were recognized as important by most participants. Furthermore, outcome measures and documentation details, such as the individual who filed the outcome (e.g., MD, nurse, or clerk), the form’s completion, and required signatures, were deemed essential by 56.2% of experts. Measures including the disability rating scale (66.7%), pain scales (60.0%), and quality of life assessments (73.3%) were also widely acknowledged as essential (Figure 3).

### 3.4 Optional and Excluded Variables

**FIGURE 4:**
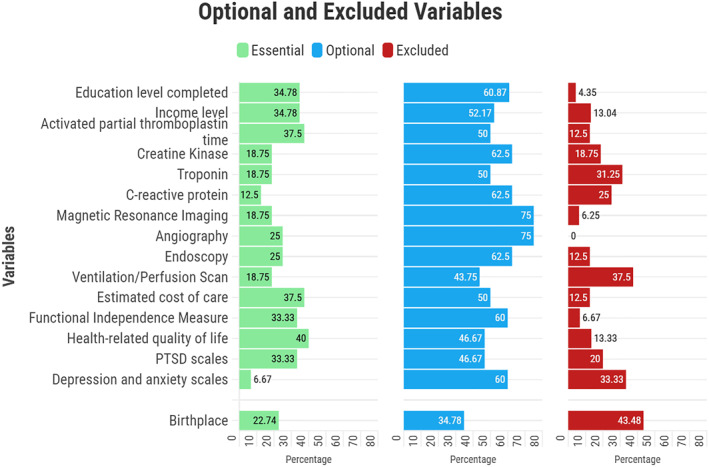
Variables that reached consensus as optional or were excluded in the second round of the Delphi survey.

In the second round of the survey, several variables were deemed optional, such as education level completed (60.9%), income level (52.2%), and certain advanced imaging modalities, such as angiography (75.0%), endoscopy (62.5%), ventilation/perfusion scan, and magnetic resonance imaging (MRI), along with some specific laboratory tests. Additionally, variables related to the cost of care and outcome measures were also considered optional by most experts, including the Functional Independence Measure, health‐related quality of life, post‐traumatic stress disorder (PTSD) scale, and scales for depression and anxiety. Birthplace (43.5%) was identified as the only variable to be excluded from the trauma registry (Figure 4).

## 4 Discussion

This Delphi survey offers clear insights into what is essential for a trauma registry in LMICs. It highlights a shared understanding among experts from all income nations about the need for thorough and standardized trauma documentation. The strong agreement on several key elements indicates that the variables are widely recognized as critical for the registry's objectives, reflecting shared expert opinion and enhancing the validity of the findings, supporting earlier recommendations for developing context‐specific registries [8]. Trauma registries in HICs, such as the National Trauma Data Bank (NTDB) and Trauma Quality Improvement Program (TQIP), include around 80 to over 110 data fields per patient, supporting comprehensive benchmarking and risk adjustment [10]. In contrast, trauma registries in LMICs often use streamlined datasets of 20–40 variables to ensure feasibility and data completeness [11, 12]. Our Delphi process identified 56 essential variables, representing a balanced and context‐adapted dataset. This number reflects expert consensus on what is essential for effective trauma care and feasible to collect in LMICs. Notably, some variables are composite and may require disaggregation at the point of implementation to align with clinical documentation practices. The high consensus on patient identification, demographic details, and injury‐related variables is consistent with other research highlighting the significance of these critical components to monitoring trauma system trends, supplying benchmarking data, and identifying injury trends, including distribution by age, geographic location, and cause of injury [7, 13].

The consensus to include prehospital data, including arrival time, mode of arrival, and transfer status, reflects the growing understanding of the trauma care continuum in LMICs and the significance of this data for evaluating timeliness and appropriateness of care [6] as well as assessing and enhancing interventions in the prehospital setting, which have been shown to reduce mortality rates [14, 15]. In addition, the strong agreement on patient outcome data, including discharge and mortality, aligns with the primary goals of trauma registries in evaluating outcomes and quality of patient care [7, 16]. The consensus on including complications and disability evaluation demonstrates a holistic approach to trauma care assessment. The significant burden of trauma associated with road traffic accidents in many LMICs is shown by the inclusion of data on these incidents [17]. This information can inform local public health policy on the local circumstances in the area.

Interestingly, experts in the second round recognized other variables as essential, such as measures of injury severity, such as the ISS and KTS, and some of the variables rated as “essential” may be used for calculating the KTS and RTS, highlighting their importance in trauma assessment and registry development. Although severity scores allow comparison with national and international norms, they have major limits in terms of interobserver reliability, validity, and interpretation [18, 19, 20, 21, 22]. The KTS provides a simplified version of the Revised Trauma Score (RTS) and ISS that is dependable in trauma registries in underdeveloped nations such as Uganda [12]. It was created especially for settings with low resources.

The addition of quality‐of‐life evaluations and rehabilitation data indicates a shift in trauma care toward long‐term outcome evaluation, as it provides a comprehensive understanding of patient recovery beyond the initial treatment phase. This approach helps identify areas for improvement in acute and rehabilitative care, informs evidence‐based policy and resource allocation, and promotes patient‐centered care. Additionally, it enhances general population health and addresses recovery inequities [23]. The designation of certain variables as optional, such as advanced imaging modalities and specific laboratory tests, likely reflects the lack of resources in many LMICs [24].

Multiple studies have highlighted the problem of standardizing trauma data collection [13, 24]. A standardized trauma registry in LMICs could align with regional, national, and international systems, allowing comparative analyses of trauma care and outcomes. This enables quality improvement and optimal care of injured patients while addressing local needs [3]. However, establishing and maintaining such registries in resource‐constrained settings necessitates substantial financial, human, and technological investment as well as staff training [25]. In addition, the extensive amount of data collected and stored means that trauma registry operations are labor‐intensive and expensive. Incomplete data in some registries continues to limit their value, and therefore [26, 27, 28], it is important to have a balance between what is ideal and what is feasible. To enhance data completeness in trauma registries, it is crucial to acknowledge the limitations of recordkeeping across various clinical units. Collecting all registry data within a single clinical setting, such as the emergency department, significantly increases the likelihood of obtaining comprehensive data. Observations indicate that attrition occurs as patients transition between different clinical units, which can lead to gaps in data collection and negatively impact the quality of the registry.

However, before wide implementation, the proposed essential variables should be piloted in select LMIC settings to assess real‐world feasibility, data completeness, and local adaptability. These pilot studies are critical to validate the utility of the dataset and inform revisions based on contextual challenges. In practice, the number of routinely collected variables may be lower, as some data points—though considered essential—may not be feasible to capture consistently. This work represents an important first step, with LMIC‐based experts actively shaping a registry framework they consider most appropriate for their local context. Sustaining trauma registries also requires long‐term commitment to local ownership, investment in workforce training, and integration into national health information systems [29].

Implementing trauma registries should also account for technical factors, including hardware, software, operating systems, memory capacity, and security measures. The CGS‐developed “Amber” database is a user‐friendly web‐based application for data collection (Global Surgery Collect), management (Global Surgery Studio), and storage (cloud storage, Amber Collect) [30]. The platform operates across devices, including cellphones, by simply browsing a link, functioning offline, and seamlessly uploading data when networks are available and are not dependent on active Wi‐Fi for data entry, directly addressing the connectivity limitations commonly faced in low‐income settings [27]. It has been piloted in several countries, including Tanzania and Senegal [30]. To date, the platform has recorded over 10,500 surgical procedures in Tanzania [31]. Local stakeholders have supported the initiative for scalability to other hospitals for improving data acquisition systems. Integrating the registry’s essential variables with the Amber platform can help advance global surgery initiatives by improving information management in LMICs.

### 4.1 Limitations and Strengths

Our study has several limitations. The experts included do not represent all LMICs, which restricts the generalizability of our findings. Yet, this approach remains feasible and sustainable in many LMICs. The lower participation rate in the second round is a limitation likely due to time constraints, survey fatigue, and the absence of incentives, potentially affecting the robustness of consensus on some variables. Despite these limitations, the study validated the essential components of a functional and uniform trauma registry.

## 5 Conclusion

This study has identified the essential elements of a trauma registry in LMICs, drawing insights from experts with on‐the‐ground experience in LMICs who are well‐versed in the realities of limited resources and the core components of trauma care. This helped to ensure that the resulting trauma registry recommendations were relevant and implementable in the intended settings. Implementing such a registry, although recommended by experts, requires further piloting to assess feasibility. Ultimately, the registry serves as a foundation for quality assurance and improvement, comparisons between jurisdictions, and the development of trauma systems.

## Author Contributions

D.D.: conceptualization (equal), supervision (equal), writing – review and editing (equal). T.R.: conceptualization (equal), writing – review and editing (equal). J.G.: supervision (equal), writing – review and editing (equal). T.F.: methodology (lead), formal analysis (lead), visualization (lead), writing – original draft preparation (lead), writing – review and editing (equal). G.S.: methodology (supporting), writing – review and editing (supporting). S.M.: methodology (supporting), writing – review and editing (supporting). S.B.: writing – review and editing (supporting). I.K.: writing – review and editing (supporting). V.K.: writing – review and editing (supporting). R.B.: writing – review and editing (supporting). V.M.: writing – review and editing (supporting). All authors reviewed and approved the final version of the manuscript for publication.

## Consent

Before participating in the survey, participants were informed through the consent form, which provided comprehensive details on anonymity, confidentiality, voluntary participation, and the potential risks and benefits of the study. The objectives of the study were also outlined, highlighting the importance of the research in addressing specific issues within the field. It was clearly stated that participation was entirely voluntary, and by proceeding, participants gave their informed consent to take part in the research.

## Conflicts of Interest

The authors declare no conflicts of interest.

## Statement of Human Rights

Patients and/or the public were not involved in this research’s design, conduct, reporting, or dissemination plans.

## Data Availability

All data relevant to the study are included in the article.
